# Child, family and household characteristics associated with physical activity in Samoan children aged 3–8 years: A cross-sectional study

**DOI:** 10.1371/journal.pgph.0002886

**Published:** 2024-04-17

**Authors:** Clara R. Warmath, Courtney C. Choy, Stephen T. McGarvey, Lauren B. Sherar, Rachel L. Duckham, Christina Soti-Ulberg, Take Naseri, Muagututia S. Reupena, Dongqing Wang, Nicola L. Hawley

**Affiliations:** 1 Department of Epidemiology, School of Public Health, Brown University, Providence, Rhode Island, United States of America; 2 Department of Chronic Disease Epidemiology, Yale School of Public Health, New Haven, Connecticut, United States of America; 3 National Centre for Sport and Exercise Medicine, School of Sport and Exercise Sciences, Loughborough University, Loughborough, United Kingdom; 4 Australian Institute for Musculoskeletal Sciences (AIMSS), The University of Melbourne and Western Health, St. Albans, Victoria, Australia; 5 Ministry of Health, Apia, Samoa; 6 Lutia i Puava Ae Mapu i Fagalele, Apia, Samoa; 7 Department of Global and Community Health, College of Public Health, George Mason University, Fairfax, Virginia, United States of America; Sri Ramachandra Institute of Higher Education and Research, INDIA

## Abstract

Physical activity is a key component of many obesity prevention strategies. The aim of this analysis was to identify child, family, and household characteristics associated with parent-reported physical activity in Samoan children aged 3–8 years. Children (n = 445; 51.2% female, mean age 5.4 years) were part of an ongoing, mixed-longitudinal study of child growth, development, and wellbeing (the *Ola Tuputupua’e* cohort). Bivariate analyses and multivariate generalized linear regressions were conducted to investigate the relationship of child, family, and household characteristics with physical activity level, measured using the Netherlands Physical Activity Questionnaire (NPAQ). Children were classified as being ‘highly active’ if they had NPAQ scores in the 75^th^ percentile or above. Among the n = 111 children classified as ‘highly active’, n = 67 (60.4%) were boys. After adjusting for child, family, and household-level characteristics, hours of child sleep per night was the only variable significantly associated with odds of being highly active. Compared to children who slept less than 9 hours at night, those who slept 10–10.99 hours (OR: 5.97, 95% CI: 2.14–18.13) and 11+ hours (OR: 25.75, 95% CI: 8.14–90.12) had higher odds of being ‘highly active’. Future research should examine the mechanisms driving the relationship between nighttime sleep and physical activity among Samoan children. Intervening on sleep duration and quality may improve physical activity and, in turn, obesity risk in this setting.

## Background

The burden of childhood overweight and obesity is rapidly rising in low- and middle-income countries, and Pacific Island nations have experienced an especially dramatic rise. Between 2000 and 2019, the prevalence of overweight among children under five in Oceania rose from 4.7% to 9.4% [1]. This burden remains high throughout later childhood and adolescence, with the prevalence of overweight and obesity nearing 50% among youth under twenty in many Pacific nations [2]. Evidence from other populations indicates that early childhood overweight and obesity are risk factors for adult obesity and obesity-related morbidity [3, 4]. As such, there is an urgent need to reduce the burden of childhood overweight and obesity in the Pacific region. Samoa is one of the Pacific nations that exemplifies the early burden of childhood obesity; in an ongoing mixed longitudinal study of child growth and development obesity prevalence was shown to be 16% at age 2–4 years and 25% at 4–6 years [5].

Physical activity is a key component of many childhood obesity prevention strategies [6, 7] and may be particularly important in the Pacific region where modifying dietary intake is challenging because of issues of cost, availability, and cultural value associated with food [8]. To effectively develop and target physical activity interventions in this setting, factors associated with existing physical activity patterns in children must be understood. Data from high income settings, including two systematic reviews (both with a large majority of studies from the United States), suggest that time spent in physical activity and physical activity patterns (frequency, timing) vary by child gender, with boys generally more physically active than girls [9, 10]. Other individual factors like age, calorie intake, and socioeconomic status have been investigated, but their impact on physical activity remains equivocal with findings likely specific to community and context [10]. Significant positive associations have, however, been documented between physical activity and both sleep quality and quantity in studies conducted in multiple settings, across a wide range of economic development [11–13]. Importantly, family- and household-level factors, such as parental physical activity and attitudes towards physical activity, household environment, and family income level have also been noted to have a significant influence on child physical activity [14–19]. Though there is little evidence that child BMI is associated with physical activity, a comprehensive systematic review of child physical activity correlates found that parental BMI is associated with child physical activity levels, with children of parents with overweight/obesity being more physically active [9].

Data on child physical activity in the Pacific region are sparse and particularly limited in Samoa. A recent analysis of 3 to 7-year-old Samoan children that used data from the same cohort study identified no significant differences in accelerometer-measured physical activity levels between boys and girls [20]. This result contrasts with the body of evidence from other regions of the world, raising questions about other ways in which correlates of physical activity may differ in Samoa compared to other settings. The aim of this analysis was, therefore, to identify child, family, and household correlates of parent-reported physical activity in Samoan children aged 3–8 years.

## Methods

### Study design and participants

The *Ola Tuputupua’e* (“Growing Up”) study is a mixed longitudinal study taking place in Samoa with the goal of exploring how child, family, and household characteristics are associated with healthy growth and development during childhood. Three hundred and nineteen mother-child pairs were included in the initial wave of data collection that took place between June and August 2015. A detailed description of the cohort’s recruitment has been previously published [21]; briefly, participants were recruited, using a convenience sampling approach, from 10 villages across the Samoan island of ‘Upolu (the smaller, but more populated of the two Samoan islands) with each of the three ‘Upolu census regions approximately equally represented in the study sample [21]. The second wave of data collection (n = 459) took place between June 2017 and August 2018 with an additional village (11 in total) when children were between the ages of 3 and 8 years. In addition to 277 mother-child pairs from the first wave of data collection in 2015, 182 new mother-child pairs were recruited and included in this wave of data collection [22]. Participants were split approximately evenly between sexes; 51% of children were girls and 49% were boys.

At the time of recruitment, eligible mothers were over 18 years of age, not pregnant, and reported no severe physical or cognitive impairments. If the mother of a child recruited in 2015 was pregnant at the time of data collection in 2017/2018, the questionnaire portion of the study was administered, but no anthropometric data were taken from the mother. Eligible children at both time points were of Samoan descent (based on the maternal report of the child having four Samoan grandparents) and had no maternal-reported physical or cognitive impairments.

All study procedures were reviewed and approved by the Yale and Brown University Institutional Review Boards and the Health Research Committee of the Samoan Ministry of Health (IRB Number 2000020519 and IAA Number 18–41 959). Samoan research staff facilitated the informed consent process and all participants provided written informed parental consent for participation. Children that were seven years of age and older provided written assent, per Yale IRB protocols, while children younger than seven provided verbal assent. Consent procedures included a detailed description of the study’s purpose and procedures, the anticipated time commitment, and potential risks and benefits. All participants had a chance to ask questions and consider their participation before providing consent/assent.

### Analytic sample

**Fig 1:**
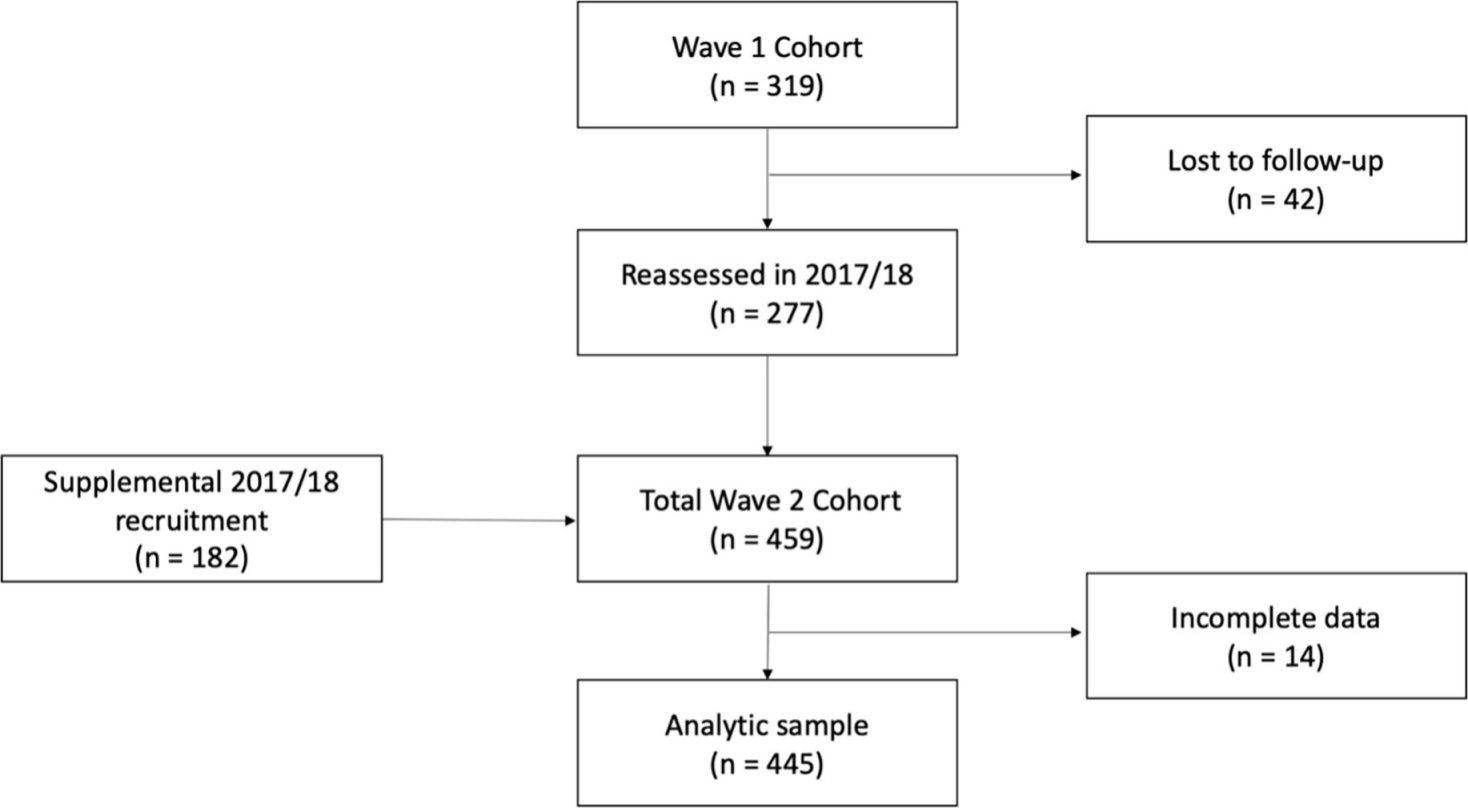
Consort diagram of analytic sample.

Our sample consisted of children who were assessed during the second wave of data collection in 2017/2018 (n = 459). Twelve children were assessed in both 2017 and 2018; this occurred if data on key variables from 2017 (for example, child anthropometric data) were incomplete and in this case, the more complete data were used. If both sets of data were complete, data from their first assessment in 2017 were used in analyses since that was when the majority of children completed survey measures. Children with incomplete physical activity data (n = 14) were excluded from these analyses. The final analytic sample included 445 children (**Fig 1**).

### Outcome assessment: Netherlands Physical Activity Questionnaire (NPAQ)

Mothers were asked to report the physical activity levels of their children using the NPAQ (**S1 File**). We chose this questionnaire for use in the first wave of cohort data collection since, at the time, it was one of the only available parental-report tools for use in 2–4-year-olds and included items that were not tied to a specific culture/setting (i.e., other tools included examples of sporting activities [basketball, gymnastics] that are not common in Samoa) [21]. The NPAQ has been used in subsequent waves and explored for its correlation with device-measured physical activity [22, 23]. Each of the seven NPAQ items has two statements situated on the opposite ends of a spectrum (e.g., “The child prefers to play inside” and “The child prefers to play outside”). Mothers were asked to score their child on a Likert scale from 1 to 5 based on how strongly their child’s behavior aligned with either statement, with 1 typically corresponding with the less active option and 5 typically corresponding to the more active option. The scales for Questions 2 and 6 were reverse coded for scoring. A total score (out of 35) was calculated by summing the scores for each question, with the highest scores theoretically indicating the highest physical activity level. We then assessed the discriminative ability of the seven NPAQ questions by examining the response frequencies to ensure that there was sufficient variation in responses (**Table 1**). We found increased variation compared to response frequencies in the same cohort two years prior [23] and determined that it was acceptable to use the full questionnaire in this age group. Given the focus on informing intervention development (specifically understanding factors associated with above-average physical activity levels) and for ease of interpretability, the child physical activity score was dichotomized into “highly active” and “not highly active”. Prior analyses have used a cut-point of 26 when categorizing NPAQ scores [24]. However, our goal was to identify the characteristics of “highly active” rather than merely “active” children. Thus, we chose a higher cut-point for our analysis; children were categorized as “highly active” if they scored a 29 or higher on the NPAQ, corresponding with the 75^th^ percentile of our sample.

**Table 1:** Netherland’s Physical Activity Questionnaire response frequency among Samoan children.

	1 N (%)	2 N (%)	3 N (%)	4 N (%)	5 N (%)	
Prefers to play alone	29 (6.5)	12 (2.7)	40 (9.0)	8 (1.8)	356 (80.0)	Prefers to play with others
Prefers vigorous games	191 (42.9)	39 (8.8)	134 (30.1)	23 (5.2)	58 (13.0)	Prefers quiet games
Dislikes playing sports	65 (14.6)	40 (9.0)	99 (22.2)	59 (13.3)	182 (40.9)	Likes playing sports
Is more introverted	17 (3.8)	16 (3.6)	80 (18.0)	54 (12.1)	278 (62.5)	Is more extroverted
Likes reading	81 (18.2)	85 (19.1)	137 (30).8	62 (13.9)	80 (18.0)	Dislikes reading
Likes to play outside	97 (21.8)	29 (6.5)	242 (54.4)	18 (4.0)	59 (13.3)	Likes to play inside
Less physically active compared to other children	20 (4.5)	19 (4.3)	205 (46.1)	63 (14.2)	138 (31.0)	More physically active compared to other children

*Note*. Respondents were asked to score their child from 1 to 5 based on how strongly their child’s behavior aligns with either statement with 1 typically corresponding with the less active option and 5 typically corresponding to the more active option, with the exception of questions 2 and 6 that should be reverse scored.

### Child, family, and household-level characteristics

The child, family, and household-level characteristics examined in this study were selected based on a systematic examination of literature from other settings, specifically, we chose variables in our dataset that had been consistently associated with physical activity in other settings [25, 26], and our knowledge of the local setting and community practices.

#### Child-level characteristics

Mothers were asked detailed questions about their child’s diet, screen time, sleep, and general health. Child dietary intake was measured with a 117-item food frequency questionnaire adapted for use in the Samoan context wherein mothers were asked to report how often their child had consumed each food in the past 30 days [27, 28]. Responses ranged from ‘less than once a month or never’ to ‘greater than 6 times a day’. Daily total energy and nutrient intakes were calculated by multiplying the reported consumption frequency by the nutrient content of standard portion sizes. A modern dietary pattern was then identified using principal component analysis, as previously described [22]. For this pattern with high intakes of french fries, unprocessed red meat, potatoes, cereals, noodles, and fruit juice, factor scores were dichotomized as “low” or “high” according to the sample median to describe levels of adherence to a modern diet among children. We summed maternal-reported time spent watching TV, playing on a computer, or playing on a phone each day to calculate total child screen time per day (an average across week and weekend days). Finally, mothers were asked to estimate the usual number of hours their child sleeps each night.

Duplicate height and weight measurements were taken for each child and averaged for use in analyses. Weight-for-age *Z*-score (WAZ), height-for-age *Z*-score (HAZ), and BMI-for-age *Z*-score (BMIZ) were calculated based on WHO Child Growth Standards (for children under 5) and WHO Child Growth References (for children ages 5–8 years) [29, 30]. A fingerstick capillary blood sample was collected to assess hemoglobin levels, an indicator of anemia, which is particularly prevalent across the Pacific (including Samoa) and may have implications for energy and activity levels in children [31, 32]. Anemia was defined as having a hemoglobin level less than 110 g/L in children under 5 and less than 115 g/L in children 5 or older [33].

#### Family-level characteristics

Mothers were also asked to provide information about themselves, including their age, highest level of education, employment status, and how they perceived their own health and weight. Maternal physical activity was estimated using the Global Physical Activity Questionnaire (GPAQ), a physical activity questionnaire validated for use in developing countries [34–36]. Due to the large proportion of mothers that reported zero minutes of moderate-to-vigorous physical activity (MVPA), maternal physical activity was dichotomized into mothers with zero minutes of MVPA per day and those reporting any amount of MVPA. Maternal height and weight measurements were used to calculate BMI. Polynesian BMI cut-offs, chosen for their sensitivity to a higher ratio of lean mass to fat mass in this population, were used to categorize maternal BMI [37]. Mothers’ perceptions about their weight and general health were also evaluated. Mothers were asked if they perceived their weight to be ‘much too heavy’, ‘moderately heavy’, ‘just right’, ‘too light’, or ‘don’t know’ and if they perceived their health to be ‘excellent’, ‘very good’, ‘good’, ‘poor’ or ‘very poor’. Paternal age and highest level of education were also document. For consistency with prior analyses, maternal and paternal age were both categorized as ‘18–24.9 years’, ‘25–39.9 years’, or ‘≥40 years’ [20–23].

#### Household-level characteristics

Household ownership of eighteen consumer durable items (such as a TV and refrigerator) was used to calculate a household asset score, with higher scores indicating greater socioeconomic resources. Categorization was based on previous use of this measure in the Samoan population [21]. Household food security was measured with the Latin American and Caribbean Food Security Scale (ELCSA), an experience-based survey composed of 16 questions that asks mothers to report if they or their child have experienced manifestations of food insecurity in the previous six months [38, 39]. Each affirmative answer was summed to calculate a total score out of 16. We categorized this score into 3 groups: food secure (ELCSA = 0), food insecure/very food insecure (ELCSA = 1–10), and severely food insecure (11–16) [38]. Mothers’ perception of the community spirit in their village (a possible proxy for children’s participation in community-led organized sports) was assessed with a 5-point Likert scale question with response options ranging from ‘very weak’ to ‘very strong’. Mothers were also asked to report the total annual household income for the prior year using categories in line with the Samoan census [40] and the number of children living in the household. Census region of village residence was recorded to approximate the degree of urbanization with Apia Urban Area being the most urban, Northwest Upolu being ‘periurban’, and Rest of Upolu being the least urban.

### Statistical analyses

We first described the characteristics of our sample stratified by sex. To evaluate differences between girls and boys, we used t-tests for continuous variables with normal distributions, Wilcoxon–Mann–Whitney tests for continuous variables with non-normal distributions, and Chi-Square or Fisher’s Exact tests for categorical variables.

Bivariate analyses of maternal-reported child physical activity with selected child, family, and household-level characteristics were conducted using Pearson’s correlations and proportion tests to identify potential covariates for inclusion in logistic regression models. Given that there were minimal differences in associations between boys and girls in exploratory analyses, we chose not to stratify our multivariate analyses by sex to maximize sample size.

Multivariable regression of child physical activity on child-, family-, and household-level characteristics was performed using logistic regression models. Child sex, child age, and census region of residence were included in all models regardless of their significance to ensure that the observed associations were not confounded by these fixed sample characteristics. Other covariates were included if their association with child physical activity was significant at the 0.10 level in bivariate analyses. Variables were added into the model in three groups: child-level variables, family-level variables, and then household-level variables. Model fit was evaluated using Bayesian information criteria (BIC), with lower BIC indicating better model fit. Odds ratios were reported for all variables in logistic regressions; for continuous variables, odds ratios should be interpreted as the increase in odds per 1 unit increase in the respective variable. All analyses were conducted in R using version 3.6.1 [41].

## Results

### Sample characteristics

The average age of children in our sample was 5.4± 1.0 years and a large majority of children were enrolled in school (73.3%). Over 90% of both mothers and fathers had completed at least high school (**Table 2**). No significant differences between boys and girls were observed in the distributions of child age, school enrollment, maternal and paternal demographics, household asset score, or census region (**S1 Table**).

**Table 2:** Proportion of highly active children by child, family, and household-level characteristics.

		Highly active (NPAQ ≥ 29)	
	n (%) or mean ± SD	% or rho^1^	*p*-value
**Child**			
Sex			*0*.*007*
Male	217 (48.8)	30.9	
Female	228 (51.2)	19.3	
Age	5.4 ± 1.0	-0.05	*0*.*290*
Weight-for-age *Z-*score	0.4 ± 1.2	0.1	*0*.*003*
Height-for-age *Z-*score	-0.2 ± 1.2	0.1	*0*.*203*
BMI Z-score			*0*.*197*
<1	289 (65.8)	22.5	
1–1.99	114 (26.0)	29.0	
≥2	36 (8.2)	33.3	
Screen time (minutes)			*0*.*146*
0	32 (7.2)	28.1	
≤60	121 (27.2)	19.0	
≤120	126 (28.3)	32.5	
≤180	100 (22.5)	22.0	
>180	66 (14.8)	24.2	
Modern diet			*0*.*021*
High	220 (50.5)	20.0	
Low	224 (49.5)	29.9	
Total caloric intake (cal)	5695.5 ± 5790.0	-0.1	*0*.*016*
Sleep (hours)			*<0*.*001*
<9	109 (24.7)	10.1	
9–9.99	90 (20.4)	8.9	
10–10.99	104 (23.6)	23.1	
≥11	138 (31.3)	49.3	
School enrollment			*0*.*068*
Yes	319 (26.7)	21.9	
No	116 (73.3)	31.0	
Anemia			*0*.*278*
Yes	115 (73.3)	20.9	
No	316 (26.7)	26.6	
**Family**			
Mother’s BMI			*0*.*716*
<27	38 (9.1)	21.1	
27–31.99	111 (26.5)	24.3	
≥32	270 (64.4)	26.7	
Mother’s self-perception of health			*0*.*002*
Excellent	257 (58.0)	31.1	
Very good	54 (12.2)	13.0	
Good	102 (23.0)	16.7	
Poor	30 (6.8)	16.7	
Mother’s self-perception of weight			*<0*.*001*
Much too heavy	64 (26.9)	12.5	
Moderately heavy	110 (14.5)	15.5	
Just right	119 (24.9)	25.2	
Too light	12 (2.7)	0.0	
Don’t know	137 (31.0)	39.4	
Mother’s physical activity (average daily minutes of MVPA)			*0*.*011*
0	295 (66.3)	28.8	
≥1	150 (33.7)	17.3	
Mother’s age			*0*.*210*
18–24.99	36 (8.1)	36.1	
25–39.99	280 (62.9)	25.0	
≥40	129 (29.0)	21.7	
Father’s age			*0*.*237*
18–24.99	13 (3.0)	38.5	
25–39.99	217 (49.3)	27.2	
≥40	210 (47.7)	21.9	
Mother’s education			*0*.*861*
Elementary school or less	33 (7.4)	24.2	
High school	316 (71.0)	24.4	
College/university or higher	96 (21.6)	27.1	
Father’s education			*0*.*918*
Elementary school or less	23 (7.0)	26.1	
High school	230 (70.7)	25.7	
College/university or higher	77 (2.3)	23.4	
**Household**			
Number of children in home			*0*.*740*
1 (participating child)	24 (5.4)	29.2	
2–3	144 (32.5)	22.2	
4–5	148 (33.4)	25.0	
≥6	127 (28.7)	27.6	
Income			*0*.*662*
<10,000 tala	330 (75.3)	25.5	
10,000–29,999 tala	80 (18.3)	21.3	
≥30,000 tala	28 (6.4)	28.6	
Food security			*0*.*013*
Food secure	239 (76.2)	28.6	
Food insecure/Very food insecure	162 (13.9)	22.8	
Severely food insecure	44 (9.9)	9.1	
Asset score			*0*.*200*
0–2	114 (25.7)	27.2	
3–5	86 (19.4)	18.6	
6–8	114 (25.7)	30.7	
≥8	130 (29.3)	22.3	
Region			*0*.*505*
Apia Urban Area	143 (32.1)	23.1	
Northwest Upolu	156 (35.1)	28.2	
Rest of Upolu	146 (32.8)	23.3	
Village spirit			*<0*.*001*
Weak/Average	164 (37.0)	15.2	
Strong	107 (24.2)	39.3	
Very strong	172 (38.8)	25.0	

^1^ Rho calculated using Pearson’s correlation test for continuous variables. Categorical variables evaluated using tests for equality of proportio

### Associations of child, family, and household-level variables with physical activity

In bivariate analyses (**Table 2**), hours of sleep per night were significantly positively associated with a child being “highly active” (*p* < 0.001). Child sex was also strongly associated with physical activity; 30.9% of boys were categorized as highly active compared to only 19.3% of girls (*p* = 0.007). A positive correlation was also observed between physical activity and WAZ (rho = 0.1, *p* = 0.003). Among children with a higher adherence to a modern diet, fewer were classified as “highly active” compared to children with low adherence (20% vs 29.9%, *p* = 0.021). Similarly, a negative association was observed with total caloric intake (*p* = 0.016). Physical activity did not significantly differ by child age, child BMI, or minutes of screen time per day.

Several maternal characteristics were associated with physical activity. As mothers’ perceived themselves to be less healthy, child physical activity decreased (*p* = 0.003). Fewer children of mothers who perceived their weight as being ‘much too heavy’ or ‘moderately heavy’ were “highly active” compared to children of mothers who perceived their weight as ‘just right’ or responded that they didn’t know (*p* < 0.001). Maternal physical activity was also strongly associated with child physical activity level. Interestingly, children of mothers that did not report daily moderate-to-vigorous physical activity (MVPA) were more active than children of mothers who reported daily MVPA (*p* = 0.011). Other family-level characteristics such as mothers’ BMI, age, and education level and fathers’ age and education level were not associated with child physical activity. Of the household-level characteristics examined, food security (p = 0.013) and strong village spirit (*p* < 0.001) were associated with higher child physical activity level.

### Multivariable analyses

Multivariable regression was conducted to further investigate the relationship of correlates with physical activity level while controlling for child sex, child age, and census region (**Table 3**). Based on the bivariate analyses described above, child-level covariates included weight-for-age z-score, adherence to a modern dietary pattern, total caloric intake, sleep, and school enrollment; family-level included perceived maternal health and weight and maternal physical activity; household-level covariates were food security and perceived village spirit. Before adjusting for other covariates, the odds of being classified as “highly active” were 48% lower for females compared with males (*p* = 0.004). As variables were added to the model, the association of child sex with physical activity level was attenuated (Model 1 (fixed study characteristics only): OR = 0.52, 95% CI 0.34–0.81; Model 4 (fixed study characteristics with child, family, and household-level variables): OR = 0.62, 95% CI 0.37–1.85). In Models 2 and 3, WAZ also contributed significantly to the prediction of physical activity level (Model 2 (fixed study characteristics with child-level variables): OR = 1.27, 95% CI 1.03–1.59; Model 3 (fixed study characteristics with child and family-level variables): OR = 1.30, 95% CI 1.03–1.65). Consistent with bivariate analyses, sleep was strongly associated with physical activity level in all models (Model 4, *p* < 0.001). Compared to children who slept less than 9 hours at night, those who slept 10–10.99 hours (Model 4, OR: 5.97, 95% CI: 2.14–18.13) and 11+ hours (Model 4, OR: 25.75, 95% CI: 8.14–90.12) had higher odds of being ‘highly active’.

**Table 3:** Logistic regression models of child physical activity on child, family, and household-level characteristics.

	1				2				3				4			
	*B* (SE)	OR	Lower	Upper	*B* (SE)	OR	Lower	Upper	*B* (SE)	OR	Lower	Upper	*B* (SE)	OR	Lower	Upper
**Constant**	0.13 (0.68)	1.14	0.30	4.39	-1.06 (0.87)	0.35	0.06	1.89	-1.01 (1.01)	0.37	0.05	2.61	-1.73 (1.20)	0.18	0.02	1.85
**Number of Observations**	445				426				422				420			
**Pseudo R** ^**2**^																
Hosmer and Lemeshow	.024				.171				.193				.199			
Cox and Snell	.027				.175				.193				.198			
Nagelkerke	.040				.259				.287				.296			
**FIXED VARIABLES**																
**Female**	-0.65** (0.22)	0.52	0.34	0.81	-0.58* (0.25)	0.56	0.34	0.92	-0.52* (0.26)	0.59	0.35	0.99	-0.48 (1.20)	0.62	0.37	1.85
**Age (years)**	-0.20 (0.12)	0.82	0.65	1.04	-0.13 (0.15)	0.88	0.65	1.18	-0.08 (0.16)	0.93	0.68	1.27	-0.08 (0.16)	0.92	0.67	1.04
**Region** (ref: Rest of Upolu)																
Northwest Upolu	0.28 (0.27)	1.33	0.78	2.26	0.18 (0.31)	1.20	0.65	2.23	0.05 (0.32)	1.06	0.56	1.99	0.14 (0.33)	1.15	0.60	2.20
Apia Urban Area	0.05 (0.28)	1.05	0.60	1.83	0.07 (0.33)	1.08	0.57	2.04	-0.06 (0.34)	0.94	0.48	1.84	0.03 (0.35)	1.03	0.51	2.07
**CHILD-LEVEL VARIABLES**																
**Weight-for-age *Z*-score** ^1^					0.24* (0.11)	1.27	1.03	1.59	0.26* (0.12)	1.30	1.03	1.65	0.21 (0.12)	1.23	0.97	1.57
**Modern diet**					-0.34 (0.27)	0.71	0.42	1.20	-0.32 (0.27)	0.72	0.42	1.23	-0.30 (0.28)	0.74	0.43	1.28
**Total caloric intake(cal)** ^1^					0.00 (0.00)	1.00	1.00	1.00	0.00 (0.00)	1.00	1.00	1.00	0.00 (0.00)	1.00	1.00	1.00
**Hours of sleep** (ref: <9)																
9–9.99					-0.18 (0.52)	0.84	0.29	2.29	0.32 (0.58)	1.38	0.43	4.23	0.66 (0.62)	1.94	0.56	6.55
10–10.99					1.04* (0.41)	2.82	1.29	6.49	1.47** (0.49)	4.35	1.70	11.83	1.79*** (0.54)	5.97	2.14	18.13
≥11					2.17*** (0.38)	8.77	4.31	19.28	2.99*** (0.49)	19.84	6.64	64.62	3.32*** (0.61)	25.75	8.14	90.12
**School enrollment**					-0.42 (0.31)	0.66	0.36	1.22	-0.50 (0.32)	0.61	0.32	1.14	-0.45 (0.32)	0.64	0.34	1.21
**FAMILY-LEVEL VARIABLES**																
**Mother’s self-perception of health** (ref: excellent)																
Very good									-0.51 (0.50)	0.60	0.21	1.54	-0.36 (0.52)	0.70	0.24	1.85
Good									-0.15 (0.38)	0.86	0.41	1.79	-0.13 (0.39)	0.88	0.40	1.87
Poor									-0.50 (0.62)	0.61	0.16	1.91	-0.67 (0.64)	0.51	0.13	1.68
**Mother’s self-perception of weight** (ref: just right/too light)																
Much too heavy									-0.61 (0.51)	0.54	0.19	1.41	-0.49 (0.53)	0.61	0.21	1.67
Moderately heavy									0.05 (0.41)	1.05	0.47	2.34	0.08 (0.43)	1.09	0.47	2.53
Don’t know									-0.58 (0.44)	0.56	0.23	1.30	-0.24 (0.53)	0.78	0.27	2.17
**Mother’s physically active**									0.46 (0.38)	1.58	0.75	3.34	0.53 (0.39)	1.69	0.80	3.64
**Mother’s age** (ref: 18–24.99)																
25–39.99									-0.56 (0.45)	0.57	0.24	1.39	-0.55 (0.45)	0.58	0.24	1.42
≥40									-0.89 (0.49)	0.41	0.16	1.07	-0.84 (0.49)	0.43	0.17	1.13
**HOUSEHOLD-LEVEL VARIABLES**																
**Food security** (ref: Food secure)																
Food insecure/Very food insecure													0.12 (0.42)	1.12	0.49	2.60
Severely food insecure													-0.03 (0.65)	0.97	0.24	3.26
**Village spirit** (ref: weak/average)																
Strong													-0.82 (0.47)	0.92	0.36	2.30
Very strong													0.44 (0.40)	1.56	0.71	3.46

*Note*. OR: odds ratio, SE: standard error, Lower/Upper: 95% confidence interval.

*** indicates *p* < 0.001

** indicates *p* < 0.01

* indicates *p* < 0.05.

^1^ Odds ratios for continuous variables should be interpreted as the increase in odds per 1 unit increase in the respective variable.

## Discussion

After accounting for child, family, and household-level characteristics in our models, hours of sleep per night was the only variable that was consistently associated with child physical activity level. A greater number of hours of sleep each night was associated with higher odds of a child being classified as “highly active”. Although a recent meta-analysis argues that existing data among children are not conclusive [42], several studies have associated nighttime sleep with physical activity, hypothesizing that quality sleep allows children more energy during the day to be physically active [10–12] or that physical activity (vigorous activity, in particular) promotes longer and better-quality sleep [42]. Due to the cross-sectional design of this analysis, we cannot draw conclusions about causation, nor can we address whether one or both of these pathways explain the observed association. Further longitudinal data collection is needed. While most studies noting the association between physical activity and sleep have been similarly cross-sectional [42], a randomized controlled trial among 8–11-year-old children also noted that accelerometer-measured physical activity per epoch (a standardized time interval) decreased during a period of sleep restriction and increased during a period of increased sleep [43]. In addition to the plausible biological pathways, there could also be a social explanation for this association. Mothers that encourage their child to be physically active may also be encouraging healthy sleep habits and vice versa.

Acknowledging the wide confidence intervals that surround the effect estimates, the strength of the association between sleep and physical activity observed here–after controlling for many other individual and family variables—is greater than among most other studies of children [42]. Most existing studies have been conducted in high-income settings and thus the Samoan context may be important for explaining the finding. Whereas children in high income settings may have more established sleep and physical activity schedules, children in Samoa often have more autonomy, which the authors speculate could provide more opportunity for lifestyle factors (e.g., physical activity) to influence sleep duration and vice versa [44]. With sleep holding numerous social and cognitive benefits for school-aged children, promotion of healthy sleep habits may have broad benefits beyond promotion of physical activity and–with several effective and acceptable behavioral sleep interventions available [45]–may offer an important complement to efforts to intervene on physical activity in this setting.

Although not significant in the final multivariable models, similar to studies in high-income settings [8, 9, 46, 47], we observed a significant association between female sex and lower physical activity level in bivariate analyses. This contradicts a previous study using a subset of the same sample that found no difference in accelerometer-measured physical activity between boys and girls at the same age [20]. Because the NPAQ relies on maternal report, our primary outcome is how mothers perceive their child’s physical activity rather than a more direct estimate of physical activity; in Samoan culture, masculinity and strength are valued and encouraged in boys [48] and mothers may perceive boys to be more active than girls even if their measured activity level is similar. In Samoa, household chores are traditionally heavily gendered by ‘strength’; boys’ chores are often outdoors and consist of more physical work (i.e., building, working in fields, fishing) than girls’ chores, which tend to take place in and around the house (i.e., cleaning, child care, preparing food) and may be perceived to have less energy cost [48]. The type of play children engage in also may differ by sex, with boys engaging in more active and rougher play than girls [49]. These factors likely reinforce mothers’ perceptions that boys are more active. In addition, self-reported measures are known to be subject to additional biases (recall, social desirability, among others) leading to differing estimates when compared to device-based measures. As an example, device-based physical activity monitoring detects short, sporadic bouts of ambulatory activity whereas questionnaire responses may only reflect memorable longer bouts of physical activity. The NPAQ also requires parents to assess physical activity relative to their child’s peers (e.g. “more or less physically active than children of the same age” rather than asking for an estimate of minutes of physical activity).

Many of the other child, family, and household characteristics that were significantly associated with child physical activity in bivariate analyses were associated in expected directions: children who adhered more closely to a modern diet were also perceived to be less physically active; fewer children from households with severe food insecurity were “highly active” while a greater proportion of children from communities with strong or very strong community spirit were “highly active”). The positive association observed between child WAZ and parent-reported physical activity may be explained either by community/cultural values that equate larger physical size with strength and fitness [50, 51] or by messaging about the health risks of obesity; mothers of larger children may be more actively promoting physical activity.

In contrast, the negative association observed between maternal MVPA and child physical activity level in bivariate analyses challenges what has been documented in other populations. Prior literature comparing accelerometer-measured physical activity for both parents and children–including one study among Pacific Island families in New Zealand–has reported strong positive associations between parent/child physical activity levels.^14-16^ The opposite finding seen in our cohort could be a result of the large proportion of mothers in our cohort that reported zero minutes of MVPA (66.3%, **Table 2**) reducing the discriminative ability of this variable. This association may also be influenced by maternal perceptions; mothers who perceive themselves to be less active may be more likely to perceive their child as “highly active” in comparison.

The most notable strength of this study is that it is the first analysis to broadly examine factors associated with physical activity in Samoan children. This work is in line with growing efforts to encourage child physical activity in the Pacific region. The cross-sectional design of our study, however, limits our ability to draw conclusions about causation or the mechanisms driving the observed associations. Particularly for the relationship between nighttime sleep and physical activity in children, there were high odds ratios and wide confidence intervals that warrant further investigation. In addition, our findings may not be generalizable to the Samoan population due to the cohort recruitment strategy. The sample distribution was intended to equally represent each of the three census regions, overrepresenting children from the Apia Urban Area, rather than the actual population distribution. Finally, although our analysis used estimates of physical activity, relative to peers, from a proxy-report questionnaire, rather than device-based measurement of time spent physically active, the utility of such questionnaires has been previously established [52] and the NPAQ has previously been compared to accelerometer measurements in this community [23].

Future research should focus on investigating the mechanisms driving the relationship between sleep and physical activity in childhood. This would require sleep, physical activity, and health-related factors to be assessed at different time points for the same children so that a temporal association could be established. In addition, NPAQ data should be complemented by device-based measures of physical activity so that factors associated with more objectively measured time spent in physical activity, physical activity patterns, and intensity may be explored to inform future research and interventions. Finally, investigation of physical activity correlates in other age groups is necessary to determine if these associations continue throughout later childhood. While there is more work to do, the results presented here suggest an opportunity for preventative obesity/non-communicable disease interventions in this population–by promoting healthy sleep habits, in particular, it may be possible to increase physical activity levels in this population and improve overall health.

## Supporting information

S1 Checklist Inclusivity in global research. (DOCX)

S1 File Samoan translation of the Netherlands Physical Activity Questionnaire. (DOCX)

S2 File Samoan abstract. (DOCX)

S1 Table Child, family, and household-level characteristics by child sex. (DOCX)
