# Supplementary material for: Child, family and household characteristics associated with physical activity in Samoan children aged 3–8 years: A cross-sectional study
Source: PLOS Glob Public Health. 2024 Apr 17;4(4):e0002886. doi: 10.1371/journal.pgph.0002886 (PMC11023467; doi:10.1371/journal.pgph.0002886)
Supplement: S1 File — (DOCX) [file pgph.0002886.s002.docx]

**Supplementary File 1. Samoan translation of the Netherlands Physical Activity Questionnaire**

**Mo fesili o i lalo ifo, fa’amolemole li’o le numera e fa’amatala ai [igoa] i le ono masina ua tuana’i atu nei. Fa’ata’ita’iga, afai e fiafia [igoa]  e taalo to’atasi nai lo’o le taalo faatasi ma isi tamaiti, li’o le numera 3 mo le fesili muamua. Afai e fiafia lou alo e taalo ma isi tamaiti nai lo’o le taalo na o ia, li’o le numera 5.**

For the following questions, please circle the number that best describes [name] during the past six months. For example, if [name] preferred to play alone as often as he/she preferred to play with other children, circle the number 3 for the first question. On the other hand, if he or she almost always preferred playing with other children, rather than alone, circle the number 5.

Almost always About Equal Almost always

**Tele o taimi Isi taimi Tele o taimi**

**E fiafia e taalo na o ia** 1 2 3 4 5 **E fiafia e taalo ma isi tamaiti**

Prefers to play alone Prefers to play with other children

**E fiafia taaloga e malosi** 1 2 3 4 5 **E fiafia taaloga filemu**

(e.g. tag, kickball) (e.g. board games)

Prefers vigorous games Prefers quiet games

**E lē fiafia e taalo i ta’aloga** 1 2 3 4 5 **E fiafia e taalo i ta’aloga**

(e.g. soccer, basketball) Likes playing sports

Dislikes playing sports

**E filemu** 1 2 3 4 5 **E tautalatala**

(e.g. quiet, reserved) (e.g. outgoing)

Is more introverted Is more extroverted

**E fiafia e faitau** 1 2 3 4 5 **E lē fiafia e faitau**

Likes to read Dislikes reading

**E fiafia e taalo i fafo** 1 2 3 4 5 **E fiafia e taalo i totonu o le fale**

Likes to play outside Likes to play inside (home/school)

**E lē fiafia a gaioi e pei o isi** 1 2 3 4 5 **E fiafia e gaioi e pei o isi**

**tamaiti e tutusa o latou tausaga** **tamaiti e tutusa o latou tausaga**

Less physically active compared More physically active compared

to children of the same age to children of the same age
