# Supplementary material for: Child, family and household characteristics associated with physical activity in Samoan children aged 3–8 years: A cross-sectional study
Source: PLOS Glob Public Health. 2024 Apr 17;4(4):e0002886. doi: 10.1371/journal.pgph.0002886 (PMC11023467; doi:10.1371/journal.pgph.0002886)
Supplement: S2 File — (DOCX) [file pgph.0002886.s003.docx]

**Supplementary File 2. Samoan Abstract**

**FA’AMATALAGA**

Tala’aga:  O le fa’amalositino o le tasi lea o vaega autu mai I le tele o aua e puipui ai oe mai I le tino puta. O le faamoemoega o lenei au’ili’iliga,o le fa’ailoa lea o uiga o tamaiti,o le aiga,ma le aiga atoa e fesootai ma fa’agaioiga faaletino,na lipotia e matua I tamaiti Samoa o tausaga o le 3-8 tausaga.

Auala:  Tamaiti (n=445, 51.2% tama’ita’i, lona uiga o le matua 5.4 tausaga) e tatau ona faaauau ,o se vaega o su’esu’ega faifaipea fefiloi o le tuputupua’e ,atina’e ma le soifua manuia o tamaiti,(Ola Tuputupua’e ). Na faalua ona faia su’esu’ega ina ia su’esu’e ai le va o tamaiti,aiga,ma le aiga atoa i le tulaga o le fa’amalositino,ma fuaina e faaaoga ai le fesili mai a le Netherlands Physical Activity questionnaire (NPAQ). O tamaiti na fa’avasegaina e “maualuga le to’aga” pe a maua ni togi o le NPAQ i le 75th pasene pe sili atu.

I’uga: I le n=111 o tamaiti ua fa’avasegaina le maualuga o le gaioi, n=26 (60.4%) o tama. I le fetu’unaiga mo tamaiti, o aiga, ma tulaga o aiga, itula e momoe ai tamaiti i le po na o le pau lea o le fesuiaiga e matua’i feso’ota’i ma faigata o le maualuga o le gaioi. Fa’atusa i tamaiti o lo’o momoe itiiti ifo i le 9 itula i le po, ma i latou e momoe i le 10-10.99 itula (po’o le 5.97, 95% CI: 2.14-18.13) ma le 11+ itula (po’o le 25.75, 95% CI: 8.14-90.12) e faigata tele i le tulaga o le gaioi tele.

Fa’aiuga: O su’esu’ega i le lumana’i e tatau ona su’esu’eina faiga o auala e fa’atautaia ai le feso’otai’ga i le va o le moe i le po ma gaioiga o le tino i tamaiti Samoa. O le fa’alavelave i le umi o le moe ma le lelei e mafai ona fa’aleleia atili ai le fa’agaioiga fa’aletino, ma, i le isi itu, e ono a’afia ai i le tino puta i lea tulaga.
