# Supplementary material for: Child, family and household characteristics associated with physical activity in Samoan children aged 3–8 years: A cross-sectional study
Source: PLOS Glob Public Health. 2024 Apr 17;4(4):e0002886. doi: 10.1371/journal.pgph.0002886 (PMC11023467; doi:10.1371/journal.pgph.0002886)
Supplement: S1 Table — (DOCX) [file pgph.0002886.s004.docx]

**Supplementary Table 1.** Child, family, and household-level characteristics by child sex

| **Characteristics** | **Total**  **(n=445)** | **Girls**  **(n=228)** | **Boys**  **(n=217)** | ***p***^1^ |
| --- | --- | --- | --- | --- |
|  | **N(%) or mean ± SD** | **N(%) or mean ± SD** | **N(%) or mean ± SD** |  |
| **Child** |  |  |  |  |
| Age (years) | 5.4 ± 1.0 | 5.3 ± 0.9 | 5.4 ± 1.0 | *0.323* |
| Attending school ^2^ |  |  |  | *0.520* |
| Yes | 319 (73.3) | 167 (74.9) | 152 (71.7) |  |
| No | 116 (26.7) | 56 (25.1) | 60 (28.3) |  |
| **Family** |  |  |  |  |
| Mother’s age (years) |  |  |  | *0.975* |
| 18-24.9 | 36 (8.1) | 18 (7.9) | 18 (8.3) |  |
| 25-39.9 | 280 (62.9) | 143 (62.7) | 137 (63.1) |  |
| ≥40 | 129 (29.0) | 67 (29.4) | 62 (28.6) |  |
| Father’s age (years) ^2^ |  |  |  | *0.467* |
| 18-24.9 | 13 (3.0) | 7 (3.1) | 6 (2.8) |  |
| 25-39.9 | 217 (49.3) | 104 (46.4) | 113 (52.3) |  |
| ≥40 | 210 (47.7) | 113 (50.4) | 97 (44.9) |  |
| Mother’s education level |  |  |  | *0.903* |
| Elementary school or less | 33 (7.4) | 18 (7.9) | 15 (6.9) |  |
| High school | 316 (71.0) | 162 (71.1) | 154 (71.0) |  |
| College/university or higher | 96 (21.6) | 48 (21.1) | 48 (22.1) |  |
| Father’s education level ^2^ |  |  |  | *0.210* |
| Elementary school or less | 23 (7.0) | 15 (8.6) | 8 (5.2) |  |
| High school | 230 (69.7) | 115 (65.7) | 115 (74.2) |  |
| College/university or higher | 77 (23.3) | 45 (25.7) | 32 (20.6) |  |
| **Household** |  |  |  |  |
| Asset score (range 0-18) ^2^ |  |  |  | *0.188* |
| 0-2 | 114 (25.7) | 58 (25.6) | 56 (25.8) |  |
| 3-5 | 86 (19.4) | 51 (22.5) | 35 (16.1) |  |
| 6-8 | 114 (25.7) | 50 (22.0) | 64 (29.5) |  |
| 8+ | 130 (29.3) | 68 (30.0) | 62 (28.6) |  |
| Income ^2^ |  |  |  | *0.644* |
| <10,000 tala | 330 (75.3) | 171 (76.0) | 159 (74.6) |  |
| 10,000-29,999 tala | 80 (18.3) | 38 (16.9) | 42 (19.7) |  |
| ≥30,000 tala | 28 (6.4) | 16 (7.1) | 12 (5.6) |  |
| Census region |  |  |  | *0.396* |
| Rest of Upolu | 143 (32.1) | 68 (29.8) | 75 (34.6) |  |
| Northwest Upolu | 156 (35.1) | 79 (34.6) | 77 (35.5) |  |
| Apia Urban Area | 146 (32.8) | 81 (35.5) | 65 (30.0) |  |

^1^ *P*-values for t-test for continuous variables with normal distribution, Wilcoxon-Mann-Whitney test for continuous variables with non-normal distribution, Chi-Square test or Fisher’s Exact for categorical variables.

^2^ Counts do not sum to total; 10 children missing school attendance data, 5 children missing father’s age data, 115 children missing father’s education data, 1 child missing asset score data, and 7 children missing from household income data.
